# Online Information About Side Effects and Safety Concerns of Semaglutide: Mixed Methods Study of YouTube Videos

**DOI:** 10.2196/59767

**Published:** 2025-04-08

**Authors:** Andy Wai Kan Yeung, Fabian Peter Hammerle, Sybille Behrens, Maima Matin, Michel-Edwar Mickael, Olena Litvinova, Emil D Parvanov, Maria Kletecka-Pulker, Atanas G Atanasov

**Affiliations:** 1 Oral and Maxillofacial Radiology, Applied Oral Sciences and Community Dental Care, Faculty of Dentistry, The University of Hong Kong Hong Kong China; 2 Ludwig Boltzmann Institute Digital Health and Patient Safety, Medical University of Vienna Vienna Austria; 3 Department of Anaesthesia, Intensive Care Medicine and Pain Medicine, Medical University of Vienna Vienna Austria; 4 Institute of Genetics and Animal Biotechnology of the Polish Academy of Sciences, Jastrzebiec Magdalenka Poland; 5 Department of Management and Quality Assurance in Pharmacy, National University of Pharmacy of the Ministry of Health of Ukraine Kharkiv Ukraine; 6 Department of Translational Stem Cell Biology, Research Institute of the Medical University of Varna Varna Bulgaria; 7 Institute for Ethics and Law in Medicine, University of Vienna Vienna Austria

**Keywords:** YouTube, semaglutide, social media, Ozempic, Wegovy, Rybelsus, safety, knowledge exchange, side effects, online information, online, videos, health issues, drugs, weight loss, assessment, long-term data, consultation

## Abstract

**Background:**

Social media has been extensively used by the public to seek information and share views on health issues. Recently, the proper and off-label use of semaglutide drugs for weight loss has attracted huge media attention and led to temporary supply shortages.

**Objective:**

The aim of this study was to perform a content analysis on English YouTube (Google) videos related to semaglutide.

**Methods:**

YouTube was searched with the words semaglutide, Ozempic, Wegovy, and Rybelsus. The first 30 full-length videos (videos without a time limit) and 30 shorts (videos that are no longer than 1 minute) resulting from each search word were recorded. After discounting duplicates resulting from multiple searches, a total of 96 full-length videos and 93 shorts were analyzed. Video content was evaluated by 3 tools, that is, a custom checklist, a Global Quality Score (GQS), and Modified DISCERN. Readability and sentiment of the transcripts were also assessed.

**Results:**

There was no significant difference in the mean number of views between full-length videos and shorts (mean 288,563.1, SD 513,598.3 vs mean 188,465.2, SD 780,376.2, *P*=.30). The former had better content quality in terms of GQS, Modified DISCERN, and the number of mentioned points from the custom checklist (all *P*<.001). The transcript readability of both types of videos was at a fairly easy level and mainly had a neutral tone. Full-length videos from health sources had a higher content quality in terms of GQS and Modified DISCERN (both *P*<.001) than their counterparts.

**Conclusions:**

The analyzed videos lacked coverage of several important aspects, including the lack of long-term data, the persistence of side effects due to the long half-life of semaglutide, and the risk of counterfeit drugs. It is crucial for the public to be aware that videos cannot replace consultations with physicians.

## Introduction

Public health information has traditionally been disseminated through printed media. However, with the rise of online social media platforms [1], the internet has become increasingly influential in spreading information and misinformation, notably during the COVID-19 pandemic [2-5]. Safety concerns in health care are frequently discussed on social media platforms such as YouTube (Google) [2,6].

Currently, obesity and being overweight are urgent health issues that reduce quality of life and increase the risk of cardiovascular diseases, type 2 diabetes mellitus, cancers, and reproductive system disorders, among others [7]. While lifestyle changes are essential for managing obesity, many people struggle with adherence [8]. Consequently, medical organizations are developing clinical guidelines for the long-term use of pharmacological therapy for obesity in adults. For instance, the American Gastroenterological Association recommends the use of pharmacological agents (strong recommendation, moderate certainty evidence) for adults with obesity or overweight who have insufficient results from lifestyle changes [8].

Unfortunately, some patients take their own antiobesity medication based on social media information, which can be dangerous without professional guidance. In particular, many people have watched YouTube videos on weight loss. It was found that the 98 most viewed weight loss videos on YouTube were viewed more than 365 million times in total [9]. In recent years, the injectable antidiabetic drug with weight loss property, branded Ozempic, has gained significant attention on platforms such as TikTok (ByteDance) and YouTube. Between 2018 and 2023, online searches for Ozempic surged in the United States [10]. Celebrity endorsements have driven its popularity, with 100 TikTok videos garnering over 70 million views [11]. However, this trend is concerning as many social media posts focused on the off-label use of Ozempic for weight loss, ignoring the potential health hazards [12]. Analyses of Reddit posts and social media comments have revealed discussions about off-label uses, struggles with insurance coverage, interest in compounded formulations, and unwanted side effects such as insomnia, anxiety, and depression [13,14].

Semaglutide, the active ingredient in Ozempic, was developed in 2012 to treat type 2 diabetes [15]. It is a glucagon-like peptide-1 (GLP-1) receptor agonist. GLP-1 receptors are expressed in many organs (pancreas, gastrointestinal tract, heart, brain, kidneys, lungs, and thyroid). This is associated with the pleiotropy and benefits of semaglutide in type 2 diabetes mellitus, weight loss, and cardioprotection. It can lower blood sugar levels through numerous means, including increasing insulin production, inhibiting glucagon secretion, and slowing gastric emptying [16]. Semaglutide is marketed as Ozempic and Rybelsus for treating diabetes and as Wegovy for chronic weight management. Ozempic and Wegovy are injectable, whereas Rybelsus is an oral tablet.

In 2017, the SUSTAIN (Semaglutide Unabated Sustainability in Treatment of Type 2 Diabetes) 1 trial demonstrated that weekly injections of semaglutide significantly improved body weight as well as glycated hemoglobin (HbA_1c_) levels in type 2 diabetes patients [17]. The STEP (Semaglutide Treatment Effect in People with obesity) 1 trial published in 2021 showed that semaglutide, combined with lifestyle changes, significantly reduced body weight in overweight or obese nondiabetic patients [18]. These trials and their subsequent trials facilitated the United States Food and Drug Administration (FDA) to approve injectable semaglutide for treating diabetes and weight management. Besides, semaglutide was also approved in Europe [19]. Meanwhile, the PIONEER (Peptide Innovation for Early Diabetes Treatment) 1 trial published in 2019 found that daily oral semaglutide, versus placebo, significantly improved HbA_1c_ levels in type 2 diabetes patients managed by diet and exercise [20]. This supported the FDA approval of oral semaglutide to treat diabetes.

During the development of semaglutide (*la semaine* in translation from French, “week”), researchers sought to increase its duration of action. The half-life of oral semaglutide is approximately 1 week [21]. Ozempic (in strengths of 2 mg/1.5 mL, 2 mg/3 mL, 4 mg/3 mL, and 8 mg/3 mL), Rybelsus (3, 7, and 14 mg), and Wegovy (0.25 mg/0.5 mL, 0.5 mg/0.5 mL, 1 mg/0.5 mL, 1.7 mg/0.75 mL, and 2.4 mg/0.75 mL) have different dosages depending on the treatment purposes and patient characteristics. Ozempic, Rybelsus, and Wegovy are prescription medicines [22]. Common side effects include a slowdown in the digestive process from the stomach, nausea, and vomiting, which can be mitigated by gradually increasing the dose. Semaglutide is associated with increased risks of pancreatitis, gallbladder disease, and retinopathy, including vitreous hemorrhage and vision loss [8,23]. Besides, the rapid decrease in glucose levels can also worsen retinopathy in type 1 diabetes patients. Semaglutide is contraindicated in patients with a personal or family history of medullary or multiple thyroid cancer or endocrine neoplasia syndrome type 2. Serious side effects include abdominal pain, constipation, diarrhea, nausea, vomiting, dizziness, cholelithiasis, cholecystitis, acute myocardial infarction, gastroenteritis, and suicidal ideation [8].

The use of semaglutide, particularly the famous Ozempic, by nonsevere overweight individuals might pose safety issues [24]. Off-label drug use is legal and common, though it means a drug is being used for an unapproved indication or population, at an unapproved dosage, or via an unapproved route of administration [24,25]. Off-label users may have a higher safety risk if they obtain semaglutide via online vendors or beauty spas without a proper medical consultation [26]. With this background, this study aimed to investigate whether YouTube videos mentioned or discussed the side effects and safety concerns of semaglutide. It was hypothesized that full-length videos should be more informative than shorts (limited to 1 minute) and that full-length videos uploaded by YouTube-verified health source channels should be more informative than their counterparts. For readers’ information, a video coming from YouTube-verified health source channels would have an information panel underneath the video stating that it comes “from a channel with a licensed doctor (or health professional)” in a particular country, such as the United States.

## Methods

### Data Source and Search Strategy

On January 5, 2024, a search was performed on YouTube for semaglutide videos in English. Using Google Chrome with Incognito mode, YouTube was searched with the words semaglutide, Ozempic, Wegovy, and Rybelsus, respectively. For each search word, the first 30 full-length videos (videos without a time limit) and 30 shorts (videos that are no longer than 1 minute) resulting from the search, sorted by relevance, were recorded. The number of 30 videos was chosen according to a recent study, which claimed that very few YouTube users searched beyond the 33rd video [27]. After discounting duplicates and excluding unsuitable videos, a total of 96 full-length videos and 93 shorts were analyzed.

### Outcome Measures

We recorded the basic video metrics, such as the duration, number of views, number of comments, number of likes, number of channel subscribers, and the age of the video since upload (number of days until March 14, 2024). The readability and sentiment of the video transcripts were assessed. The quality of video content was evaluated. Further details are described in further sections.

### Data Extraction

To evaluate the readability and overall sentiment, video transcripts were generated and analyzed. To evaluate the quality of video content, the entire videos were watched and analyzed.

The video transcripts were generated by Whisper (with the “large” version, edition 20231117), an artificial intelligence automatic speech recognition system developed by OpenAI [28]. It has been used in previous research to transcribe educational videos [29] and had the best performance compared with similar automatic transcription tools [30]. The readability of the transcripts was evaluated by the Flesch Reading Ease (FRE) score [31], calculated via an online platform (Readability Formulas website). In brief, the score ranged from 0 to 100, with 90 to 100 being very easy, 0 to 29 being very difficult, and 60 to 69 being standard. Meanwhile, the sentiment of the transcripts was evaluated by ChatGPT 3.5, an artificial intelligence large language model developed by OpenAI that has been demonstrated to be very effective in sentiment analysis across multiple languages [32]. Referring to the method by Fu et al [32], the prompt was set as “Is the sentiment of this text positive, neutral, or negative? Respond with the sentiment label only.” The “temperature” of the ChatGPT model was set at 0 to ensure the consistency of the answers with the least creativity. Temperature is a variable that changes the degree of randomness of the output generated by the model [33].

Next, the quality of video content was evaluated by 3 tools, Global Quality Score (GQS) [34], Modified DISCERN [35], and a custom checklist. Manual evaluations were independently performed by two authors (AWKY and AGA). Disagreements were resolved through mutual discussion. During these evaluations, the overall audiovisual content of the videos, not merely limited to verbal narrative, was examined. The GQS is a 5-point Likert scale designed to evaluate online health information. A score of 1 means “poor quality, poor flow, most information missing, not at all useful for patients,” whereas a score of 5 means excellence and high usefulness for patients [34]. Meanwhile, the Modified DISCERN contains 5 evaluative items and gives 1 point for every positive answer and 0 points for negative answer. It was designed to evaluate YouTube videos on health care information [35]. The 5 items are as follows: (1) Are the aims clear and achieved? (2) Are reliable sources of information used? (3) Is the information presented balanced and unbiased? (4) Are additional sources of information listed for patient reference? (5) Are areas of uncertainty mentioned? For readers’ information, the Modified DISCERN is based on an original version of DISCERN, which was designed to evaluate written health information and used a 5-point Likert scale to answer 15 questions plus an overall rating [36]. Since GQS and Modified DISCERN could only give a more general evaluation of the videos, a custom 12-point checklist was devised to evaluate the video content based on some specific aspects of side effects and safety concerns related specifically to semaglutide.

The custom 12-point checklist was compiled by the authors’ team with reference to Smits and Van Raalte [37]. It recorded whether the following contents were mentioned in the videos or not: (1) form of application (injection, exception: oral for Rybelsus); (2) safe dosage; (3) need for long-term usage, or change in lifestyle and eating habits to avoid rebound back to original weight after drug cessation; (4) serious side effects (eg, retinopathy and pancreatitis); (5) gastrointestinal symptoms (eg, nausea, diarrhea, vomiting, gastric reflux, and gastritis); (6) prevalence or frequency of such side effects; (7) increased risk of aspiration during the induction of anesthesia; (8) contraindications; (9) long half-life (7 days) so that potential side effects persist for multiple days after drug cessation; (10) lack of long-term data; (11) potential alternatives (eg, diet and bariatric surgery); and (12) risk of counterfeit drugs.

### Statistical Analysis

Following descriptive analysis of the video contents, 2-sample t tests were performed were performed to evaluate if there were significant differences between the full-length videos and shorts in terms of the mean FRE, GQS, and Modified DISCERN scores, as well as the mean number of mentioned points from the custom checklist. To supplement, the same tests were performed among the full-length videos, to evaluate if there were differences between those uploaded by YouTube-verified health source channels and those without this verification.

### Ethical Considerations

Ethical approval was not applicable, as this study only analyzed publicly available data from existing datasets, and results were presented in aggregate that did not contain any identifiable information.

## Results

**Figure 1:**
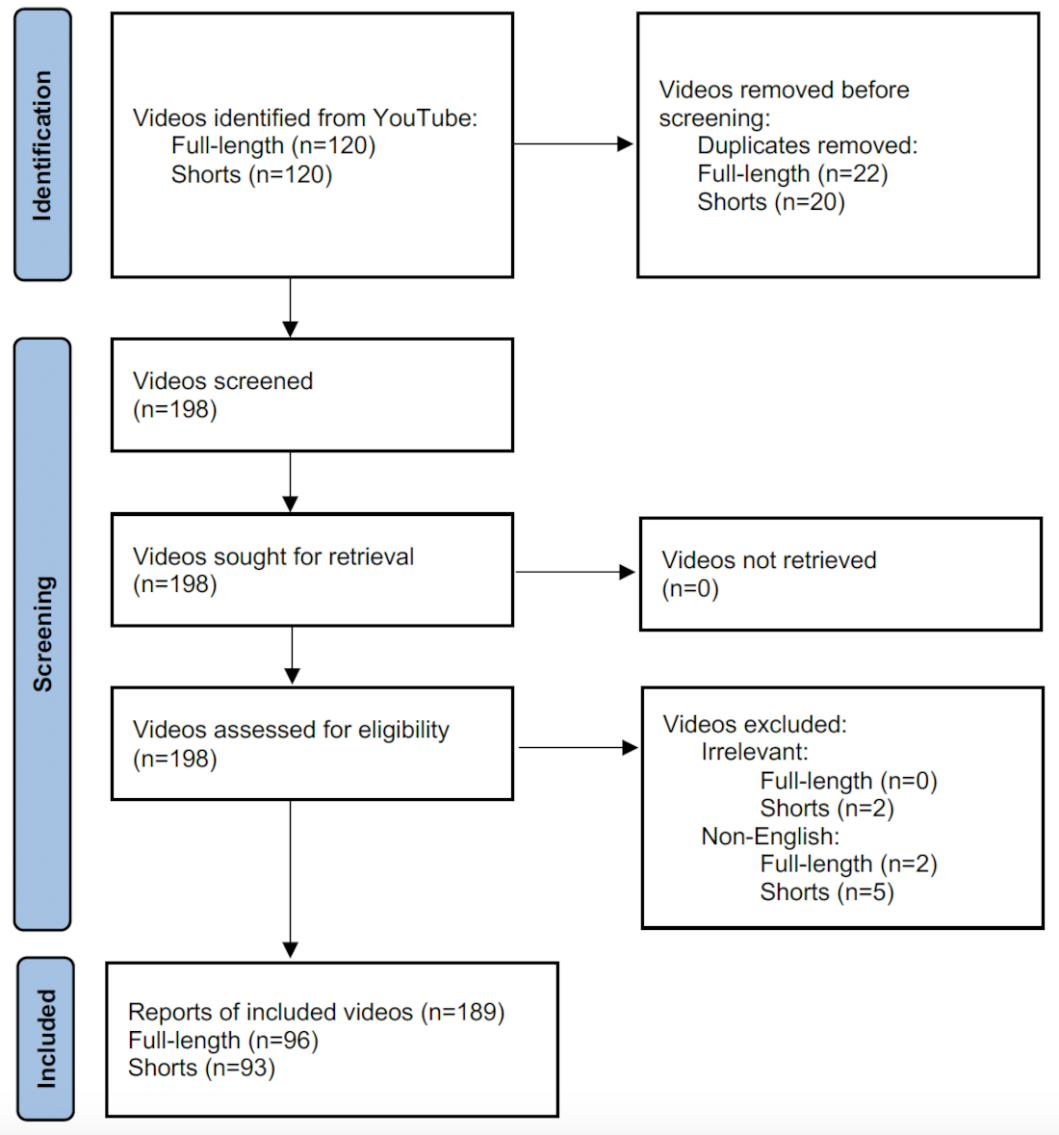
The screening process of YouTube videos on semaglutide.

**Figure 2:**
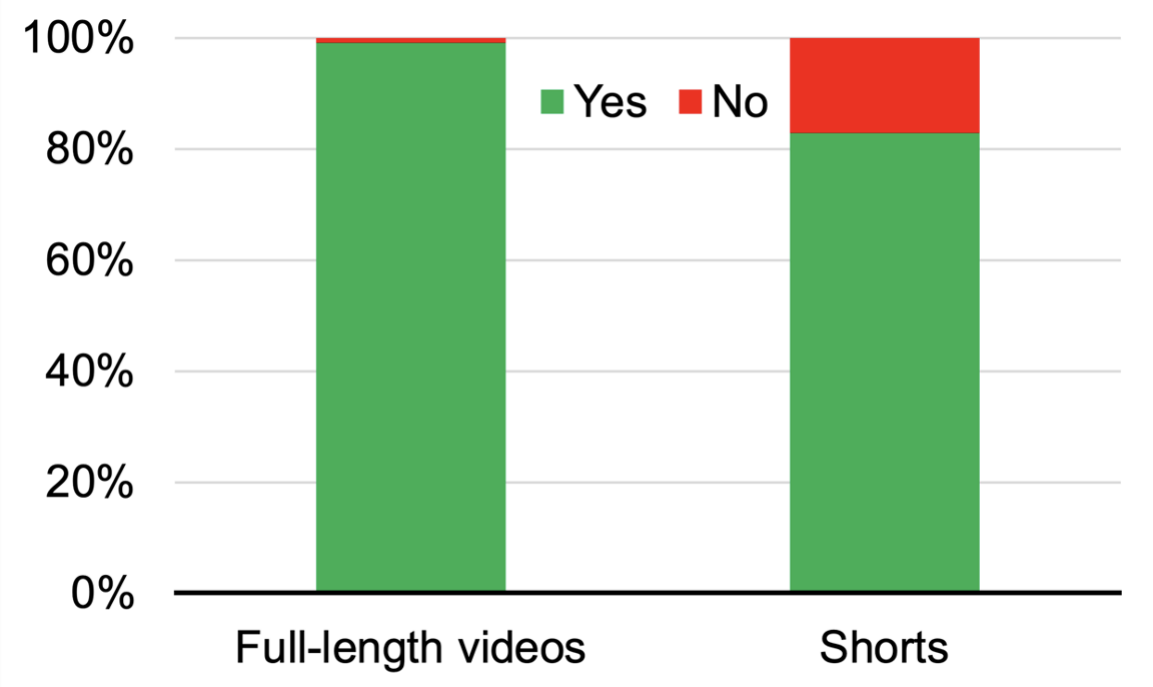
Percentage of full-length videos and shorts with a narration.

The viewing metrics and content quality between full-length videos and shorts are compared in Table 1. All 189 videos (Figure 1) were collectively viewed 45,040,855 times. There was no significant difference in the mean number of views between full-length videos and shorts (288,563.1 vs 188,465.2, *P*=.30). However, full-length videos received thrice the number of comments than shorts on average (669.6 vs 200.4, *P*=.003). Full-length videos were usually older (ie, uploaded earlier) than shorts (468.0 vs 350.0, *P*=.01). The former had better content quality in terms of GQS, Modified DISCERN, and number of mentioned points from the custom checklist than the latter (all *P*<.001). The readability of the transcripts of the 2 types of videos did not significantly differ and both were at the fairly easy level. Meanwhile, 1 full-length video did not have a narration, whereas 16 shorts played music without a narration (Figure 2). Besides, sentiment analysis showed that full-length videos mainly had a neutral tone (n=59), followed by positive (n=24) and negative (n=12) tones. One full-length video did not have a verbal narrative. Meanwhile, shorts mainly had a neutral tone (n=40), rather than positive (n=19) and negative (n=18) tones. There were 16 shorts without a verbal narrative.

Among the full-length videos, those from YouTube-verified health source channels had a higher average view count than their counterparts (Table 2), though the difference did not reach statistical significance (401,867.2 vs 258,746.3, *P*=.41). Readability analysis suggested that the transcripts from health source videos were generally less readable than their counterparts (63.7 vs 73.8, ie, standard vs fairly easy, *P*<.001). However, health source videos had a higher content quality in terms of GQS and Modified DISCERN (both *P*<.001). On average, they also contained a larger number of mentioned points from the custom checklist than their counterparts, though that difference was not significant (3.5 vs 2.7, *P*=.17)

**Figure 3:**
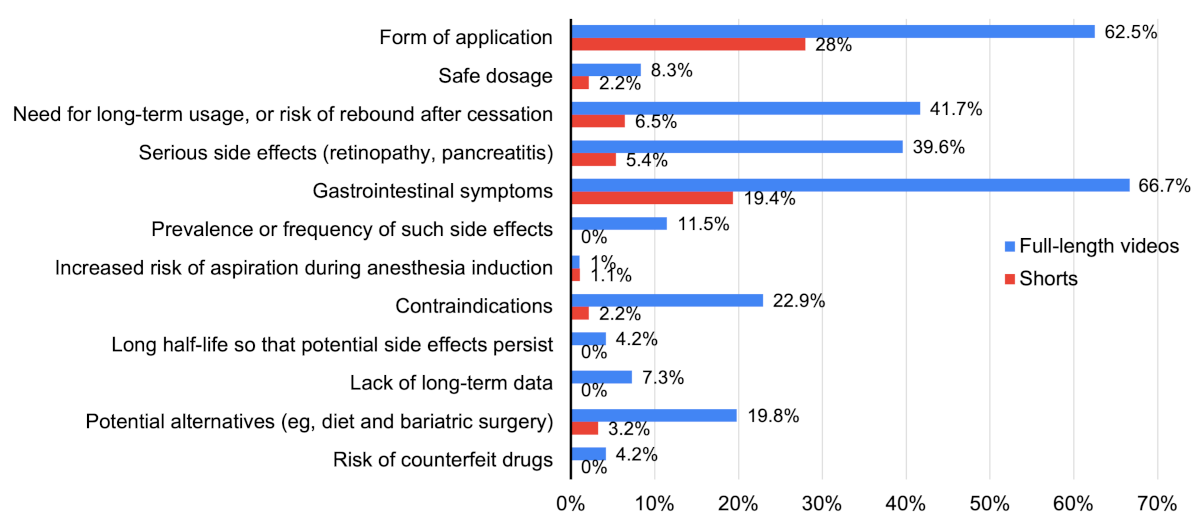
Percentage of full-length videos and shorts that covered the points from the custom checklist regarding the side effects and safety concerns of semaglutide.

Next, the reporting of the points from the custom checklist was examined. As stated in previous sections, full-length videos were generally more informative than the shorts. Gastrointestinal symptoms and form of application were the 2 mostly reported points among the full-length videos as well as the shorts (Figure 3). Among full-length videos, the most deficient aspect was the lack of mention about an increased risk of aspiration during the induction of anesthesia associated with the use of semaglutide. Only 1 full-length video (1/96) warned the audience about this potential side-effect. Less neglected aspects were the persistence of side effects due to the long half-life of semaglutide (4/96, 4%) and the risk of counterfeit drugs (4/96, 4%). On the other hand, the shorts had generally omitted several important aspects. Apart from the increased risk of aspiration during the induction of anesthesia (1/93), none of the shorts mentioned the prevalence or frequency of side effects, persistence of side effects due to the long half-life, lack of long-term data, and risk of counterfeit drugs.

**Table 1:** Viewing metrics and content quality between full-length videos and shorts.

Metric	Mean (SD)		*P* value
	Full-length videos	Shorts	
Duration (s)	610.5 (542.6)	39.0 (17.2)	<.001
View count	288,563.1 (513,598.3)	188,465.2 (780,376.2)	.30
Comment count	669.6 (1281.0)	200.4 (571.4)	.003
Like count	5363.2 (11,842.7)	7412.4 (39,692.8)	.63
Channel subscriber count	1,721,826.1 (3,500,160.1)	531,761.7 (1,783,695.8)	.004
Video age (days)	468.0 (411.4)	350.0 (152.4)	.01
Flesch Reading Ease score	71.7 (11.6)	72.9 (14.3)	.54
GQS^a^ score	2.6 (0.8)	1.1 (0.4)	<.001
Modified DISCERN	2.3 (0.8)	1.1 (0.2)	<.001
Number of content points from custom checklist	2.9 (1.7)	0.7 (0.9)	<.001

^a^GQS: Global Quality Score.

**Table 2:** Viewing metrics and content quality between full-length videos uploaded by YouTube-verified health source channels and other channels.

Metric	Mean (SD)		*P* value
	Health sources	Other channels	
Duration (s)	587.7 (664.0)	616.6 (510.9)	.83
View count	401,867.2 (733,982.5)	258,746.3 (439,684.8)	.41
Comment count	990.2 (1,631.5)	580.1 (1,163.6)	.32
Like count	10,371.5 (20,053.2)	4009.6 (8079.6)	.18
Channel subscriber count	1,873,152.0 (3,581,554.9)	1,682,003.5 (3,501,532.6)	.83
Video age (days)	630.7 (614.9)	425.1 (331.2)	.16
Flesch Reading Ease score	63.7 (13.3)	73.8 (10.2)	<.001
GQS^a^ score	3.3 (0.6)	2.4 (0.8)	<.001
Modified DISCERN	3.1 (0.6)	2.1 (0.7)	<.001
Number of content points from custom checklist	3.5 (2.3)	2.7 (1.5)	.17

^a^GQS: Global Quality Score.

## Discussion

### Principal Findings

This study found that semaglutide videos on YouTube have reached a broad audience. The 189 analyzed videos had a total view count of over 45 million. For comparison, the 98 most viewed YouTube videos on diabetic retinopathy were collectively viewed 1 million times [38]. It implied that these videos might potentially affect the perception and even health care decisions of the general public regarding the use of semaglutide. As expected, full-length videos were generally more informative than shorts. For patient education, shorts would serve better to grab the attention of the patients or make them aware of 1 or 2 particular issues related to semaglutide; whereas selected full-length videos might be more suitable to be incorporated as part of a panel discussion or public forum.

Alarmingly, the videos seldom covered the risk of counterfeit drugs. For instance, the antidiabetic drug, Ozempic, contains semaglutide that is synthesized by yeast fermentation and subsequent synthetic modification [39]. Without proper quality control of the synthetic processes, some falsified Ozempic products were found to contain contaminants such as glass particles and filler substances [39]. Meanwhile, some pharmacies would produce compounded versions of semaglutide to circumvent the patent issue. The high demand, high cost, and limited supply have led to a period of time when some patients and drug providers switched to compounded semaglutide [10]. Subsequently, the FDA stated that the compounded drugs, such as semaglutide sodium and semaglutide acetate, do not have their approval due to lack of testing, may not possess the same drug effect as semaglutide, and may even cause adverse effects (unspecified) [40,41]. Another safety issue of using compounded semaglutide is an increased risk of overdose due to suboptimal drug packaging. A recent case series reported that compounded semaglutide might be dispensed in vials instead of prefilled manufactured injection pens such as those by Ozempic and Wegovy [42]. Vials that contain large volumes of semaglutide and vials dispensed together with subpar syringes might allow for overdose much more easily during self-administration [42]. There seemed to be yet a case series on the overdose of oral semaglutide, but it would be reasoned that compounded semaglutide in the form of tablets for oral intake could have the same increased risk if each tablet did not conform to the amount of semaglutide contained in approved branded semaglutide products such as Rybelsus.

Another not uncommon risk of taking semaglutide that was often neglected by the videos was the risk of aspiration during the induction of anesthesia. One effect of semaglutide is gastroparesis, that is, reduced bowel motility and gastric emptying without any physical obstruction. This would increase the gastric volume and increase the risk of regurgitation and pulmonary aspiration of gastric contents even with the usual recommended fasting time before anesthesia [43,44]. According to a recent study, aspiration of gastric contents accounted for 5% of closed anesthesia malpractice claims in the United States during 2000-2014 [45]. Among these claim cases related to aspiration, 57% (66/115) of patients died and another 14% (16/115) suffered from permanent severe injury [45]. It implied that aspiration during anesthesia was not uncommon, and it could lead to very serious consequences. While medical organizations develop recommendations regarding the use of GLP-1 receptor agonists before operations, the optimal approach to patient data management is still being specified [46,47]. Hence, it is important for researchers and clinicians to conduct subsequent studies to optimize the fasting time and airway management strategy for patients on semaglutide who need to undergo anesthesia.

Although there have been many clinical trials on the efficacy and safety of semaglutide, most of them (if not all) had a study period of up to 2 years (104 weeks) only, such as the STEP, SUSTAIN, and PIONEER trials [37,48]. Since the use of semaglutide could last beyond 2 years and there could be a possibility of life-long usage, a lack of long-term data could mean potential risks yet to be elucidated, such as the discovery of more side effects, and potential development of drug dependence or drug resistance [49,50]. The COVID-19 vaccines may illustrate the situation of lack of long-term data. After the COVID-19 pandemic began near the end of 2019, pharmaceutical companies put huge efforts into creating vaccines that could lower the infection rate and reduce the symptoms or morbidity. By the end of 2020, at least 10 vaccines have already been introduced into the global market and authorized by governments for emergency use [51]. After several years in use, millions of people vaccinated and billions of doses administered, data have accumulated. By analyzing the retrospective data, researchers have recently found 2 new rare but potentially severe side effects of COVID-19 vaccines, namely acute disseminated encephalomyelitis and transverse myelitis [52]. This new finding may influence the decision-making of some people in the public on whether they should be vaccinated, take a booster, or choose which type of available vaccines to use. In the case of semaglutide, more data besides its weight loss and antidiabetic properties would facilitate better-informed decisions from clinicians and patients. For instance, there were cases reported on the development or recurrence of depression 1 month after taking semaglutide, which was subsequently relieved after discontinuing the drug [53]. On the other hand, a retrospective study that covered over 1.5 million patient records reported that semaglutide users had a lower risk of incident and recurrent suicidal ideation [54]. At the same time, an analysis of over 40,000 user comments posted on social media platforms has found that users of GLP-1 receptor agonists (including semaglutide) felt that the drugs have mixed effects on their mood, anxiety, and insomnia conditions [14]. Patients should be aware of the fact that many weight-loss drugs, mainly appetite suppressants, were withdrawn from the market in the past due to adverse drug reactions [55]. Therefore, in the future when long-term data become available, the safety and side effects of semaglutide could be better assessed and established.

Findings from this study echoed previous studies on Reddit content on Ozempic or semaglutide, in the sense that there is generally a lack of discussion or elaboration on the potential health risks and hazards associated with the use of Ozempic, not to mention its off-label use [12,13]. However, the risks and hazards indeed exist, such as concerns with depression and anxiety raised by social media users on YouTube and TikTok [14]. Therefore, actionable recommendations included better public health awareness campaigns to educate the public on the proper use of Ozempic or semaglutide including the potential side effects and how to manage them. Pharmaceutical companies, governments, and health authorities can organize exhibitions in shopping malls, provide easy-to-understand information on their web pages, and develop user-friendly mobile apps to engage members of the public who are interested. Future research should evaluate the effects of such activities on the knowledge level of the public on semaglutide.

This study has several limitations. First, only the first 30 full-length videos and 30 shorts were initially screened for each search word, rendering a final analysis of 189 videos. This might only represent a small proportion of the entire relevant video collection. Second, only videos in English were analyzed. The themes and foci could be different for videos produced in other languages. Third, errors might exist during computational and manual evaluations. Besides, there was not a detailed analysis of video sources, which might further enhance the findings, for example, whether videos produced by clinicians were more informative than those from general influencers. It was said that there was a tendency for the YouTube algorithm to place more “reliable” videos at the top of the search results [56], hence the presented results in this study might be biased.

### Conclusions

The 189 analyzed YouTube videos on semaglutide have attracted more than 45 million views. Full-length videos were much more informative than shorts in terms of side effects and safety issues. The analyzed videos lacked coverage of several important aspects, including the increased risk of aspiration during the induction of anesthesia, the persistence of side effects due to the long half-life of semaglutide, the risk of counterfeit drugs, and the lack of long-term data. Patients should be aware that these videos may not be comprehensive enough even if they were uploaded by YouTube-verified health source channels, and hence cannot replace the consultation from the physician, who may make tailor-made recommendations and treatment plans for each patient.

## Abbreviations

FDA: United States Food and Drug Administration
FRE: Flesch Reading Ease
GLP-1: glucagon-like peptide-1
GQS: Global Quality Score
HbA_1c_: glycated hemoglobin
PIONEER: Peptide Innovation for Early Diabetes Treatment
STEP: Semaglutide Treatment Effect in People with obesity
SUSTAIN: Semaglutide Unabated Sustainability in Treatment of Type 2 Diabetes
